# A General Aqueous Silanization Protocol to Introduce Vinyl, Mercapto or Azido Functionalities onto Cellulose Fibers and Nanocelluloses

**DOI:** 10.3390/molecules23061427

**Published:** 2018-06-12

**Authors:** Marco Beaumont, Markus Bacher, Martina Opietnik, Wolfgang Gindl-Altmutter, Antje Potthast, Thomas Rosenau

**Affiliations:** 1 Department of Chemistry, Division of Chemistry of Renewable Resources, University of Natural Resources and Life Sciences Vienna (BOKU), Konrad-Lorenz-Straße 24, A-3430 Tulln, Austria; markus.bacher@boku.ac.at (M.B.); antje.potthast@boku.ac.at (A.P.); 2 Lenzing AG, 4860 Lenzing, Austria; m.opietnik@lenzing.com; 3 Department of Materials Science and Process Engineering, Institute of Wood Technology and Renewable Materials, University of Natural Resources and Life Sciences Vienna (BOKU), A-3430 Tulln, Austria; wolfgang.gindl-altmutter@boku.ac.at; 4 Johan Gadolin Process Chemistry Centre, Åbo Akademi University, FI-20500 Åbo/Turku, Finland

**Keywords:** aqueous silanization, click chemistry, cellulose nanofibrils, nanocellulose, cellulose II gel, green chemistry, cellulose modification

## Abstract

The effective and straight-forward modification of nanostructured celluloses under aqueous conditions or as “never-dried” materials is challenging. We report a silanization protocol in water using catalytic amounts of hydrogen chloride and then sodium hydroxide in a two-step protocol. The acidic step hydrolyzes the alkoxysilane to obtain water-soluble silanols and the subsequent addition of catalytic amounts of NaOH induces a covalent reaction between cellulose surficial hydroxyl groups and the respective silanols. The developed protocol enables the incorporation of vinyl, thiol, and azido groups onto cellulose fibers and cellulose nanofibrils. In contrast to conventional methods, no curing or solvent-exchange is necessary, thereby the functionalized celluloses remain never-dried, and no agglomeration or hornification occurs in the process. The successful modification was proven by solid state NMR, ATR-IR, and EDX spectroscopy. In addition, the covalent nature of this bonding was shown by gel permeation chromatography of polyethylene glycol grafted nanofibrils. By varying the amount of silane agents or the reaction time, the silane loading could be tuned up to an amount of 1.2 mmol/g. Multifunctional materials were obtained either by prior carboxymethylation and subsequent silanization; or by simultaneously incorporating both vinyl and azido groups. The protocol reported here is an easy, general, and straight-forward avenue for introduction of anchor groups onto the surface of never-dried celluloses, ready for click chemistry post-modification, to obtain multifunctional cellulose substrates for high-value applications.

## 1. Introduction

The most abundant biopolymer, cellulose, is of enormous importance in our daily lives, simply owing to its great properties: it is a renewable, environmentally friendly, and biodegradable material. In line with recent advances in cellulose research, nanostructured celluloses come more and more to the fore for application in high-performance tailored products. Fibrillar nanocellulose is composed of high aspect-ratio nanofibers featuring unique intrinsic properties, especially with respect to rheology [1], biocompatibility [2], mechanical strength [3], optical properties [4], and morphology [5].

But one key challenge limits their potential: many beneficial properties of the nanocelluloses are (sometimes even irreversibly) lost upon drying, and the precise surface modification that is essential in many high-value applications, is difficult to achieve by means of the hydroxyl groups, in particular in an aqueous medium. Taking this issue into account, it is of major importance to find straight-forward methods to incorporate anchor groups or to introduce surface functionalities, such as fluorescent dyes [6], carbon dots [7], or proteins [8] onto the nanocellulose surface in the never-dried state. The chemical surface modification of nanocelluloses according to conventional protocols often implies time-consuming solvent-exchange, curing or drying steps, in order to allow reactions with water-sensitive reactants and to avoid water as a competing nucleophile [9]. But if one tries to abide by the principles of “green chemistry”, these solvent-exchange steps should be avoided if possible, and the same applies generally to the use of aprotic and hazardous solvents, such as dimethyl sulfoxide, *N*,*N*-dimethylacetamide or *N*,*N*-dimethylformamide. In respect of environmental compatibility, water clearly remains the solvent of choice.

An attractive way to introduce functionalities to cellulose surfaces also in aqueous media is silane chemistry, using functional trialkoxy silanes (X-Si(OR_3_) [6,10,11,12,13,14,15]. Many different triethoxy- or trimethoxy-derivatives are commercially available. They are relatively cheap, which in this case is not a limiting factor because only smaller amounts are needed, since only a surface and not a bulk modification is intended. Their fundamental chemistry is compatible with green chemistry principles, and they are generally non-toxic and biocompatible. This statement evidently refers to the siloxane part, and is of course influenced by functionalities (such as azide) appended to the silane. There have been reports on (never-dried) celluloses being functionalized using these reactants, among others with azido [12], amino [11,15], mercapto [10], and vinyl groups [13].

**Figure 1:**
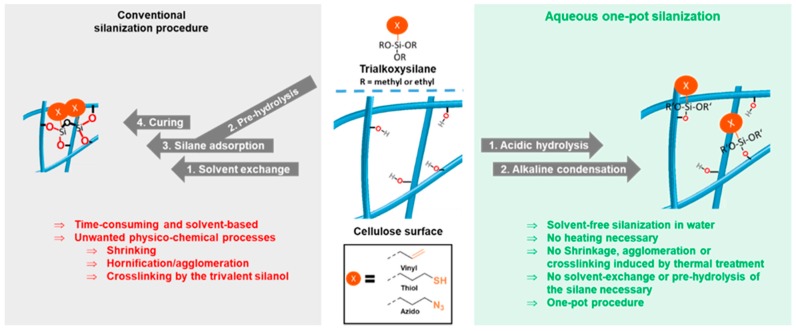
Conventional silanization procedure (**left**) and novel two-step silanization protocol (**right**): First the reaction mixture is acidified with HCl to pre-hydrolyze the alkoxysilane, then NaOH is added to induce condensation in this homogeneous system. Following this protocol, azido, vinyl, and thiol groups were introduced onto never-driedcellulose surfaces.

Conventional silane modification is conducted as schematically shown in Figure 1, left. First, the alkoxysilane is pre-hydrolyzed in an acetone/water (or alcohol/water) mixture. Meanwhile, the cellulose is solvent-exchanged to the same solvent and the hydrolyzed silanol species is added, allowing it to adsorb onto the cellulose surface. In the final step, the sample is cured to remove water and to condense the silanol with the substrate’s surface hydroxyl groups, and this way it establishes covalent linkages. This is usually the critical step, since the curing causes hornification and induces also crosslinking reactions by the trivalent silanol.

Hornification as a generic term for a decrease of external surface areas, causing closure of pores [16], shrinkage, and finally the formation of interfibrillar cellulose hydrogen bonds [17] upon the drying of cellulose. Nanocelluloses, such as cellulose nanofibrils [18,19] or nanostructured cellulose II gels possess high specific surface areas between 140 and 588 m^2^·g^−1^ [20,21,22,23,24]. Due to resulting strong interactions between individual colloid fibrils, these delicate surface structures are more prone to hornification effects than cellulose fibers [25,26]. Learning from this, conventional silanization protocols actually should not be applied to nanocelluloses, since their structure would be changed irreversibly and the product usually cannot be redispersed in water after drying, having undergone such cascade of events. Recently, we have developed a protocol to covalently link 3-azidopropyl triethoxysilane in water onto the cellulose surface [6]. In this method, the addition of a catalytic amount of sodium hydroxide induced a covalent linkage of the respective alkoxysilane to the surface at room temperature. The approach seemed quite simple and advantageous, since the reaction occurred under aqueous conditions and no heating was necessary. Consequently, the resulting azido-functionalized celluloses remained never-dried and no structural changes were induced by the surface modification.

In this contribution, we are extending this type of modification in order to render it general and applicable to more silanization agents. We envision an all-purpose toolbox to modify surfaces of never-dried celluloses and equip them with the desired functionalities for subsequent modifications in secondary reactions. The silanization, attaching the primary anchor, would thus be the first of two steps in a sequence. Evidently, it would be convenient to have a generally applicable protocol for this modification, which would work well with all kinds of celluloses and functionalized silanes. The development of this approach is presented here, and its pros and cons are critically discussed.

## 2. Results and Discussion

According to the developed two-step protocol, shown in Figure 1, right, we were able to introduce azido, vinyl, and mercapto groups onto never-dried cellulose surface in a general way, these functionalities being selected as they are frequently used in subsequent modifications (mostly click-type chemistry). This offers an easy and sustainable tool to make cellulose ready for a broad range of follow-up click chemistry; i.e., azide-alkyne [27], radical-induced thiol-ene [28], and Michael-addition thiol-ene conversions [29]. Using this modification strategy, nanocelluloses, such as cellulose nanofibrils (CNF) and cellulose II gel [22,30,31] and carboxymethylated pulp were modified.

**Figure 2:**
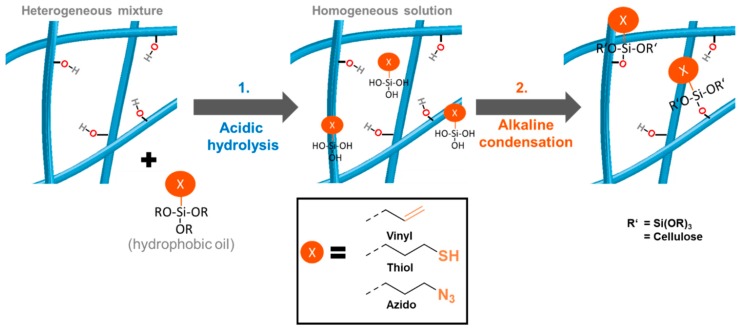
Novel two-step silanization protocol: First, the reaction mixture is acidified with dilute HCl (catalytic) to induce hydrolysis of the alkoxysilane, then dilute NaOH (catalytic) is added to induce condensation with the cellulose surface. Following this protocol, azide, vinyl and thiol functions were introduced.

Figure 2 summarizes the approach to equip the surfaces of never-dried celluloses with various functionalities—in this case vinyl, mercapto, and azido according to a general approach in aqueous medium avoiding solvent exchange, organic solvents, and drying/curing.

In contrast to conventional methods, the functionalized celluloses according to Figure 2 were obtained without the usual curing or drying step. As a consequence, no undesired physicochemical changes occur, which could have caused shrinkage, agglomeration of fibrils, decrease of external surface area, and closure of pores [16,17]. According to the protocol reported here (Figure 2), 1 wt% cellulose suspensions were used and were acidified with 8 mol% of HCl (based on cellulose anhydroglucose units (AGU)) before stoichiometric amounts (with respect to cellulosic AGU) of the respective alkoxysilane were added. Once HCl is present, the hydrophobic alkoxysilane starts to hydrolyze to hydrophilic and water-miscible silanols. After 30 min, condensation with the cellulose matrix was induced by adding 16 mol% NaOH, causing the formation of Si-O-cellulose bonds and thus the attachment of the thiol, vinyl, and azido groups, respectively. In our standard approaches, 0.6 equivalents of azido-, and mercapto silane per AGU were used alongside 0.75 equivalent of vinyl silane, due to the lower reactivity of the latter. After the HCl/NaOH treatment, the crude products were purified by washing with water and acetone (and again water if required). The acetone step is necessary to remove non-covalently bounded, adsorbed silanes from the cellulose surface. The short time of acetone-washing does not cause any physicochemical changes to the celluloses. Acetone is usually regarded as compatible with green chemistry policies—in the case of concerns in this regard, it can also be replaced by acetone/water mixtures.

In comparison to the previous protocol [6], the newly optimized approach is distinguished by the two-step sequence which improves compatibility between the reagent and matrix. The main advantage, its generality, has already been pointed out: basically, any functionality that is attachable to silane can thus be anchored onto never-dried cellulose surfaces. The three functional groups used in this work were selected because of their importance and rich history in click approaches. In addition, the silane loading of the surface was easy to control due to linear zero-order kinetics.

**Figure 3:**
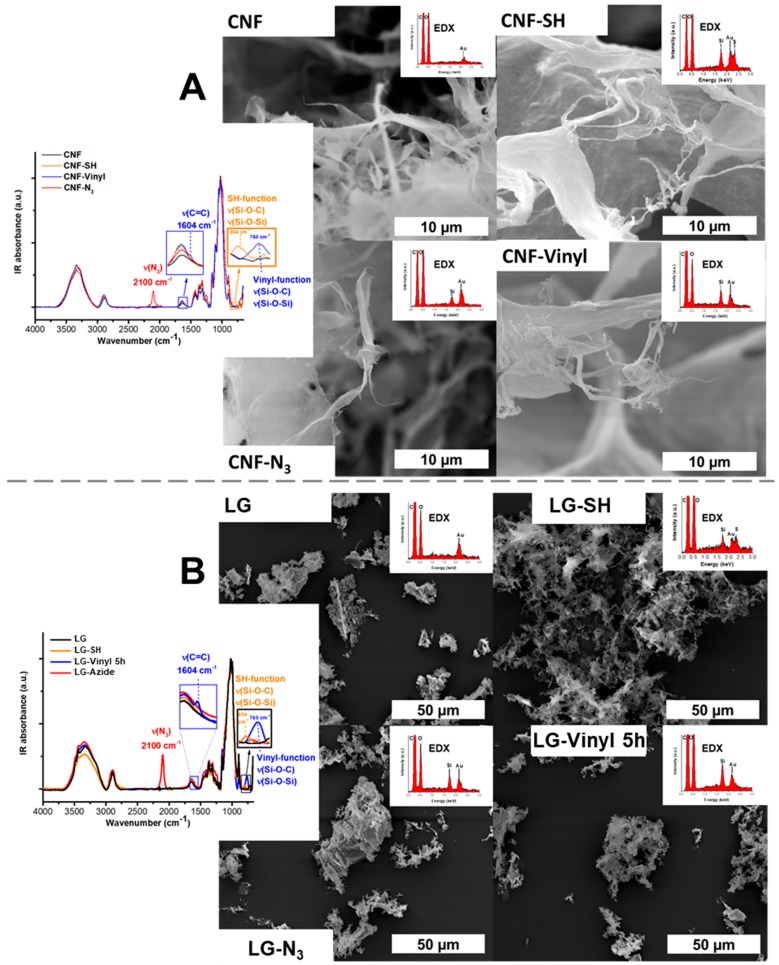
Silanization of cellulose nanofibrils (**A**) and cellulose II gel (LG, (**B**)) using mercapto-, azido- and vinyl silane as shown by infrared (IR) spectroscopy and scanning electron microscopy with energy dispersive X-ray spectroscopy (SEM-EDX). The morphology of the products was studied with SEM.

Dependent on the desired silane loading, the reaction was stopped after 2.5 h, 3 h, or 5 h. Never-dried CNF, cellulose II gel (LG or lyocell gel), and carboxymethylated pulp (cmPulp) were used in this work to demonstrate that the protocol can be used for a broad range of cellulosic substrates. The scanning electron microscopy (SEM) micrographs of the functional celluloses, the respective infrared (IR) spectra, the scanning electron microscopy with energy dispersive X-ray spectroscopy (SEM-EDX) spectra of the nanostructured celluloses and the nuclear magnetic resonance (NMR) spectra of CNF and LG are given in Figure 3 and in Figures S1 and S2 of the supporting information. The results with cmPulp as substrate are illustrated in Figure S3.

### 2.1. Introduction of Azido Groups

Azido groups are frequently used in Huisgen click chemistry with alkynes, forming 1,2,3-triazoles in a [3 + 2]-cycloaddition. The successful incorporation of azido groups into cellulosics was easily monitored by IR spectroscopy based on the characteristic azido-band at ν = 2100 cm^−1^ (Figure 3 and Figure S3). The amount of introduced groups was calculated by relating the absorbance of the azido band to a calibration curve (data from our previous publication [6] (Figure S4)).

**Figure 4:**
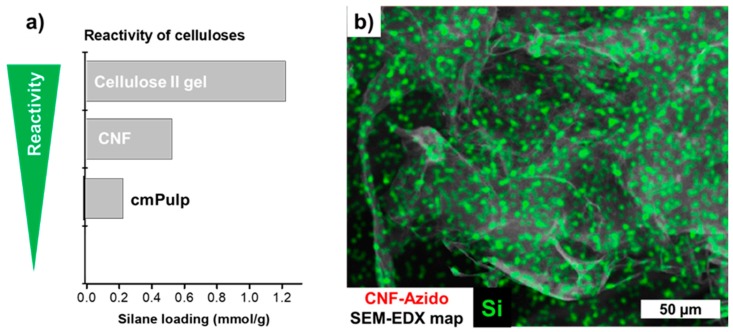
(**a**) Reactivity order of different cellulosic substrates towards 3-azidopropyl triethoxysilane. The reactivity and therefore the resulting silane loading of the nanocelluloses, cellulose nanofibrils (CNF) and cellulose II gel (LG), was significantly higher than the one of the carboxymethylated pulp (cmPulp). (**b**) The homogeneous distribution of the silane is shown by SEM-EDX (example: azido-functionalized CNF).

All substrates used were successfully equipped with azido-functionalities. The SEM micrographs in Figure 3 and Figure S3 indicate that no undesired agglomeration or other surface changes occurred upon silanization. The presence of silicon in the EDX spectra was evident. The amount of introduced azido moieties was found to be dependent on the reactivity of the substrates. As shown in Figure 4A, the csellulose II gel featured the highest reactivity, followed by CNF and pulp (1.2 mmol/g, 0.52 mmol/g, 0.23 mmol/g, respectively).

This is based on the fact that the specific surface area and reactivity increases from the microcellulose, cmPulp, towards the nanostructured materials, CNF and the cellulose II gel, as reported in the literature [6]. The difference in the azido loading of the latter pair can be reasoned by the lower molar mass (and therefore probably higher reactivity) of the cellulose II gel [30]. In comparison to the literature [6], the reactivity of cmPulp, was increased by a partial fibrillation upon stirring and silane treatment; explaining the higher silane loading of these samples (Figure S3).

The silanes reacted in a homogeneous and controlled way which was also seen by the EDX map of CNF-N_3_ in Figure 4b. Si from the azido silane was homogeneously distributed on the CNF surface and no silane particles, hypothetically formed through homopolycondensation of the respective silane, were observed.

### 2.2. Introduction of Thiol Groups

Thiol groups are often used synthons in thiol-ene click chemistry approaches. In the case of this silane, the reaction time was not extended beyond 3 h to avoid precipitation of unwanted silane particles on the cellulose substrates. The successful introduction of the thiol group was demonstrated by a combination of IR spectroscopy (Figure 3 and Figure S3) and quantitative solid state ^13^C NMR spectroscopy (Figure S1). The characteristic Si-O and Si-C stretching bands (IR) were observed, according to the literature [10,32] at ν = 804 cm^−1^ (Si-O and Si-C stretching) and the carbon resonances (NMR) at 13 and 28 ppm (Figure S1) were used to determine the silane loading by relation to the C1 peak of the cellulose AGU between 100 and 110 ppm. The reactivity of the cellulose substrates increased in the same order as in the case of the reaction with the azido silane: from the cellulose fiber substrate (cmPulp) to the nanostructured celluloses (CNF and LG), (*cf.* Table S1). Similar fibrillation phenomena as observed during azido-silanization were observed for cmPulp in the reaction with 3-mercaptopropyl trimethoxysilane. The mercapto-silane loading was controlled by varying the amount of mercapto-silane in the reaction from 0.42 to 0.97 mmol/g, LG-SH 0.3, and LG-SH (Figure S4), respectively. The high mercapto-silane loading of the microcellulose, cmPulp, i.e., 0.50 mmol/g (Table S1), emphasize the higher reactivity of this silane in comparison to vinyl-silane and azido-silane.

The correlation of the IR and NMR data resulted in a calibration curve for the silane loadings between of 0.4 to 1.0 mmol/g (Figure S4). The introduction of silane groups was confirmed once more by SEM-EDX spectra (Figure 3 and Figure S3). In contrast to the characteristic N_3_-band of the azido-modified celluloses, neither the Si-O and Si-C IR bands nor the presence of sulfur on the surface are a definite proof that free and reactive thiol groups (as -SH) are available. Especially if one considers that, in particular under alkaline conditions such as those in the condensation step, see Figure 2, thiols tend to crosslink by disulfide bridge formation [33], the presence of free -SH cannot be taken for granted. As a proof of their presence, the samples LG-SH and LG-SH 0.3 were reacted with acrylic acid by radical-induced thiol-ene reaction using 2,2-azobis(2-methylpropionamidine) dihydrochloride as the radical initiator. The carboxyl band at 1707 cm^−1^ in the IR spectra in Figure S5 proves the presence of reactive and free thiol groups in the starting material due to the successful postmodification with acrylic acid. As expected, the amount of carboxyl groups increases with increasing silane loading of the sample, from LG-SH 0.3 to LG-SH.

### 2.3. Introduction of Vinyl Groups

**Figure 5:**
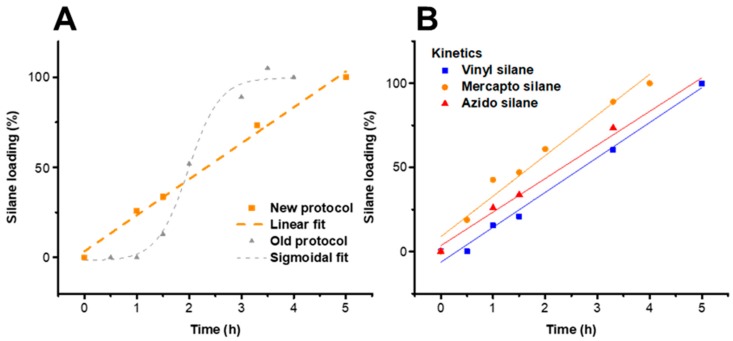
(**A**) Comparison of silanization kinetics of cellulose II gel with 3-azidopropyl triethoxysilane between novel approach and conventional literature procedure [6]. The kinetics of the two-step procedure (hydrolysis and condensation) have a linear fit (zero-order) instead of the previous sigmoidal fit. (**B**) Kinetics of the different silanization agents used in this work (substrate cellulose II gel), with the reactivity of the silanes increasing in the order vinyl, azido and mercapto silane.

Vinyl groups are often used as reactants in thiol-ene couplings, radical polymerization, or cycloadditions. The characteristic alkene stretching band at 1604 cm^1^ (Figure 3) in the vinyl-silane modified celluloses signalized an effective modification [34]. In addition, the presence of silicon atoms in the EDX spectra proved the silane on the cellulose substrates. Since the C=C IR band at 1604 cm^−1^ was partly overlapped by the band of residual adsorbed water on the cellulose [35], it was not visible in the case of rather low silane loading (vinyl-modified cmPulp). The stretching vibration of Si-O and Si-C at 760 cm^−1^ [10,34] was found more suitable to estimate and compare the silane loading of samples and was therefore also used in the kinetic study (Figure 5b and Figure S6b) and for the calibration curve (Figure S4). The calibration curve was obtained according to an approach similar to the thiol case, by relating quantitative ^13^C NMR data (vinyl peaks at 131 and 136 ppm) to the IR absorbance of the Si-O and Si-C bands at 760 cm^−1^. As shown in the kinetic plots in Figure 5b, the vinyl silane featured the lowest reactivity among the three silane reagents. This was also reflected in the low incorporation of vinyl groups onto cmPulp: the IR band of this sample had an absorbance of only 0.03%, which is approximately 70 and 300 times lower than in the case of CNF and LG, respectively. In addition, the SEM micrograph in Figure S3 shows that the reaction of cmPulp with the vinyl-silane did not cause fibrillation of the fiber surface.

### 2.4. Kinetics of Silanization

The kinetics of the three silane reactions are compared in Figure 5 for the substrate LG, by measuring the silane loading at different times during the silanization reactions (Figure S6). In all cases, the silane loading reached approximately 1.2 mmol/g, which corresponds to 100% silane loading in Figure 5B. This seems to be an approximate for a full coverage of the LG surface with the respective silanes. If this coverage is exceeded, the homocondensation of the silanols becomes more favored due to a saturation of the LG surface, resulting in heterogeneous silane particles on the cellulose surface.

From the kinetics in Figure 5B, it was obvious that the mercapto-silane featured the highest reactivity, followed by azido and vinyl silane. In contrast to the previously published protocol [6] with its sigmoidal kinetics for the introduction of azido-silane on the cellulose surface, the kinetics of the present procedure have a linear dependency. The two-step silanization reported here thus followed zero-order kinetics. This allows for an easy and reproducible control of the silane loading by variation of the reaction time as shown exemplarily by means of the samples LG-Vinyl 2.5 h and LG-Vinyl, with 0.7 and 1.2 mmol silane per gram of cellulose, respectively (*cf.* Figures S2 and S4).

### 2.5. Proof of Covalent Modification

**Figure 6:**
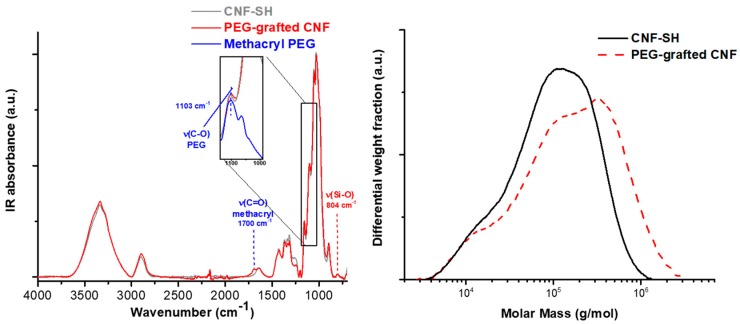
Thiol-ene grafting of methylacryl poly(ethylene glycol) (PEG) onto a cellulose nanofibril surface, IR spectrum (**left**) and the gel permeation chromatography (GPC, **right**).

In our previous publication [6], we already proved that azido-silane reacted covalently under the aqueous silanization conditions and that the silane surface coverage was not (only) an adsorption phenomenon. Additionally, Figure 6 shows the result of a postmodification of CNF-SH with mono-functionalized poly(ethylene glycol) (PEG) methacrylate by radical-induced thiol-ene, the left IR spectrum due to the carboxyl band at 1700 cm^1^ and increase in the C-O stretching vibration at 1103 cm^−1^, and the right chromatogram due to the molecular weight gain.

The molar mass distribution in Figure 6, right, proves that the PEGylated mercapto silane was covalently attached to the CNF surface. In case of an adsorbed silane, the PEG with a molar mass of about 2000 g/mol would have eluted at a different elution time closer to the salt peak. The shift of PEG-grafted CNF to higher molar masses can only be due to covalently attached substituents.

### 2.6. Stability of the Silanized Celluloses under Aqueous Conditions

**Figure 7:**
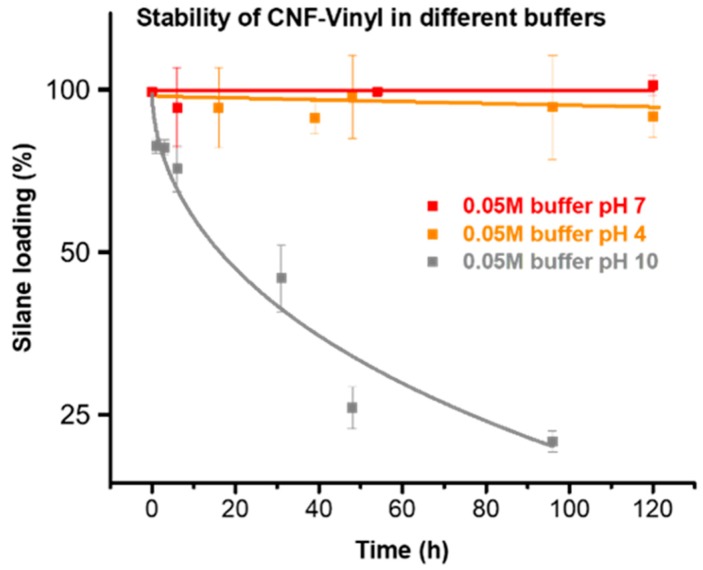
Stability of the vinyl-silane modified CNF under aqueous conditions at pH of 4, 7 and 10. The sample is stable under neutral and acidic conditions, whereas the vinyl moiety was progressively lost from the cellulose surface under alkaline conditions.

The stability of the silanized celluloses was studied under neutral (pH = 7), acidic (pH = 4), and alkaline conditions (pH = 10) using 50 mM buffers over a time of 5 days. This is shown in Figure 7 for the example of vinyl-silane modified CNF. The silane-modified cellulose can be used under both neutral and acidic conditions. No significant changes occurred at pH values 7 and 4. Under acidic conditions the silane loading was only slightly reduced from 0.43 mmol/g to 0.40 mmol/g after 4 days. Alkaline conditions, by contrast, should be avoided, since extensive hydrolysis and removal of the vinyl silane was observed. At pH 10, the silane loading was reduced from 0.43 mmol/g to 0.09 mmol/g over 4 days. Samples stored under neutral conditions were stable for months and no decrease in silane loading at all was observed. Additionally, the silanized samples proved also to be stable in organic solvents, such as acetone or *N*,*N*-dimethylacetamide.

### 2.7. An Avenue to Multi-Functional Cellulose Colloids

As demonstrated by means of the example of carboxymethylated pulp with a DS of 0.06, pre-functionalized celluloses can also be used as starting materials for the silanization protocol. A silane treatment with a combination of vinyl- and azido silane at the same time was also successful in affording multifunctional materials (Figure S7). Both avenues represent easy and readily applicable strategies to complex celluloses, which can be tuned according to their respective requirements.

**Figure 8:**
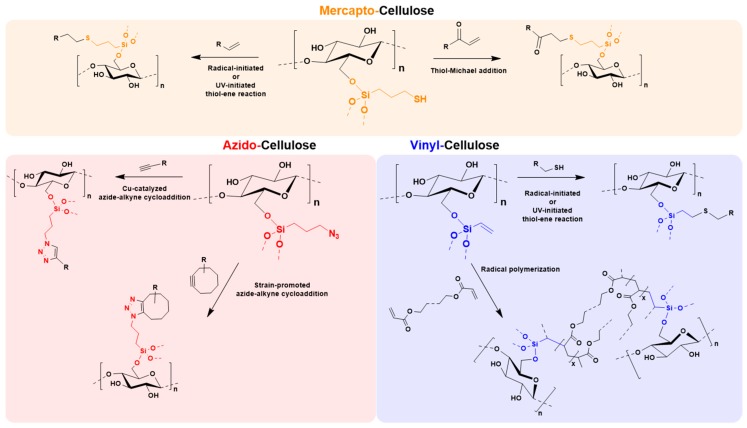
Towards a multi-purpose toolbox for modification of never-dried celluloses. Overview of possible post modification strategies for the synthesized mercapto-, azido, and vinyl-celluloses.

As shown in Figure 8, the obtained materials are useful in a variety of different click chemistry [36] approaches, among others radically or UV-induced thiol-ene processes [10,28,37], thiol-Michael reactions [29], photo-induced thiol-yne reactions [38], copper-catalyzed azide-alkyne cycloadditions [6,39,40], or strain-promoted and copper-free azide-alkyne cycloadditions [41,42]. Especially with relation to biomedical application, the mercapto, and azido silanes can be used in biorthogonal conjugations [43] by spontaneous reactions without the need of catalysts or heat, such as thiol-maleimide [44] or copper-free azide-alkyne click chemistry. Vinyl-functionalized celluloses have a great potential in 3D-printing with photocurable acrylate resins [45,46]. These few examples are just given to demonstrate the great application potential of the cellulose-modification toolbox.

## 3. Conclusions

In this contribution, we demonstrated that never-dried nano- and microstructured celluloses can be modified by a simple and general two-step aqueous silanization procedure. Mercapto-, vinyl and azido groups were introduced onto the celluloses’ surface according to the optimized protocol. In contrast to conventional silanization protocols, no curing step is necessary, therefore the functionalized celluloses remain never-dried, and no unwanted hornification and aggregation effects due to heating and drying occur. The procedure works in water, using only catalytic amounts of aqueous HCl and NaOH, thus being fully compatible with sustainable chemistry principles. The zero-order kinetics of the silanization of the cellulose surface allows a reproducible tuning of the silane loading by variation of the reaction time and amount of silane, up to a silane loading of 1.2 mmol/g.

The reactivity of the employed silanes was found to depend on the respective functional groups, increasing in the order vinyl-silane, azido-silane to mercapto-silane. As shown by EDX spectroscopy, the introduced silanes were homogeneously distributed on the surface of the functional celluloses and no Si-rich particles were observed. The modifications were stable in aqueous conditions in neutral as well as acidic environment. The protocol can also be applied to pre-functionalized celluloses (cellulose derivatives), such as carboxymethylated pulp. Alternatively, bifunctional celluloses can be obtained by simultaneous treatment with two different silanes.

As appropriate for a chemical toolbox, the generality of the approach translates into a variety of modification options and follow-up chemistry for the celluloses. Considering the sustainability and simplicity of the modification protocol in combination with the broad range of possible post-modification reactions, the potential of the presented functionalization strategy seems quite promising, and we hope that this approach will find wide acceptance among chemists concerned with celluloses and advanced functional materials.

## 4. Materials and Methods

Cellulose II gel (LG, also referred to as LENZING™ (Lenzing AG, Austria) Lyocell or lyocell gel (LENZING™ is a trademark of Lenzing AG.)) [47] with a solid content of approx. 4%, and never-dried beech sulfite dissolving pulp (KZO3, 50 wt% solid content) were provided by Lenzing AG and were stored at 8 °C. Triethoxyvinylsilane (97% purity), (3-mercaptopropyl)-trimethoxysilane (95%), 2,2-azobis(2-methylpropionamidine) dihydrochloride (97%), poly(ethylene glycol) methyl ether methacrylate solution (M_n_ = 2000 g/mol, 50 wt%) as well as all other standard chemicals were acquired from Sigma-Aldrich Chemie GmbH (Munich, Germany). 3-Azidopropyl triethoxysilane was synthesized according to [6]. Carboxymethylated pulp with a DS of 0.06 was obtained from never-dried KZO3 pulp following the protocol of [25], the degree of substitution was measured by ^1^H-NMR upon hydrolysis in D_2_SO_4_ according to the literature [48]. Cellulose nanofibrils (CNF) were produced by passing a 1 wt% pulp (never-dried KZO3) slurry 20 times through a APV-1000 (SPX Flow Inc., Charlotte, North Carolina, United States) laboratory homogenizer at 800–900 bar.

### 4.1. Silanization Protocol

#### 4.1.1. Vinyl-Modified Celluloses

To 100 mL of a 1 wt% cellulose suspension in water (1 g, 6.2 mmol) was added 1 mL of 0.5 M HCl (0.5 mmol, 8 mol%). Triethoxyvinylsilane (0.9 g, 4.0 mmol, 0.75 eq) was added and the mixture was stirred for 30 min. 0.5 M NaOH (2 mL, 1.0 mmol, 16 mol%) was added and, dependent on the desired silane loading, the reaction mixture was stirred at room temperature for different times (2.5 h or 5 h in the case of LG). Longer reaction time than 5 h caused the formation of silica deposits on the cellulose surface and should be avoided.

The gel suspension was centrifuged at 4000× *g* for 5 min and the supernatant was removed. The residue was washed (100 mL water, 50 mL acetone, 2 × 100 mL water) and centrifuged (5 min at 4000× *g*) after every washing step. Pulp was washed by filtration. The purified materials were stored as suspension in deionized water at 8 °C.

#### 4.1.2. Thiol-Modified Celluloses

The modification was performed analogue to the above protocol using 0.80 mL of (3-mercaptopropyl)-trimethoxysilane (0.76 g, 4.0 mmol, 0.63 eq) as silanization agent with only 3 h reaction time (after addition of NaOH). The sample LG-SH 0.3 was obtained using 0.32 eq of the silane.

#### 4.1.3. Azide-Modified Celluloses

The modification was done according to the above procedure with 1.0 mL of (3-azidopropyl)-triethoxysilane (1.0 g, 4.0 mmol, 0.63 eq) as the silanization agent.

#### 4.1.4. Multifunctional Celluloses

To 100 mL of 1 wt% cellulose II gel (LG) in water (1 g, 6.2 mmol) was added 1 mL of 0.5 M HCl (0.5 mmol, 8 mol%). Triethoxyvinylsilane (0.42 mL, 0.38 g, 1.8 mmol, 0.3 eq) and 0.50 mL of (3-azidopropyl)triethoxysilane (0.50 g, 2.0 mmol, 0.3 eq) were added and the mixture was stirred for 30 min. 0.5 M NaOH (2 mL, 1.0 mmol, 16 mol%) was added and the reaction mixture was stirred at room temperature for 4 h. The crude product was purified as described above for the vinyl-modified sample.

### 4.2. Kinetic Analysis

The kinetics of the silanization were studied based on the aqueous thiol-silanization protocol. 50 mL of 1 wt% cellulose II gel suspension was used as the starting material. Samples were extracted before the addition of NaOH (t = 0), after 0.5 h, 1 h, 1.5 h, 2h, 3.3 h and 5.5 h. Each sample was worked up according to the above protocol and analyzed with FTIR.

### 4.3. Functionalization of Mercapto-Celluloses

#### 4.3.1. Thiol-Ene Modification of LG with Acrylic Acid

15 mL of mercapto cellulose II gel (LG-SH, LG-SH 0.3) (1 wt%, 0.15 g, 0.93 mmol) was mixed with acrylic acid (81 µL, 85 mg, 1.2 mmol) and 2,2-Azobis(2-methylpropionamidine) dihydrochloride (0.25 g, 0.92 mmol) and heated in a water bath at 70°C overnight. The crude product was purified by a set of washing (3 × 20 mL acetone, 2 × 20 mL DI water) and centrifugation (each for 5 min at 4000× *g*). Finally, the product was freeze-dried and the success of the reaction was checked with IR spectroscopy.

#### 4.3.2. Thiol-Ene Grafting of Methacrylated Poly(ethylene glycol) onto CNF

CNF-SH (7 mL of 0.8 wt% solution, 56 mg, 0.35 mmol) was mixed with 0.4 mL of 50 wt% aqueous poly(ethylene glycol) methyl ether methacrylate solution (200 mg, M = 2 kg/mol, 0.19 mmol) and 200 mg of 2,2-azobis(2-methylpropionamidine) dihydrochloride (AAPH). The reaction mixture was heated up to 65°C in water bath for 24 h. The crude product was purified by a sequence of washing (3 × 20 mL acetone, 2 × 20 mL water) and centrifugation (each for 5 min at 4000 × g) steps. Finally, the product was freeze-dried and analyzed by IR spectroscopy.

### 4.4. Nuclear Magnetic Resonance Spectroscopy (NMR)

NMR experiments of dissolved samples were performed on a Bruker Avance II 400 instrument (Rheinstetten, Germany). The resonance frequencies were 400.13 MHz for ^1^H, 100.61 MHz for ^13^C and 79.49 MHz for ^29^Si. Solution NMR spectra were recorded in CDCl_3_ or D_2_O (both 99.8% D, Euriso-top, Saint-Aubin, France) at room temperature.

Solid state NMR experiments were performed on a Bruker Avance III HD 400 spectrometer (resonance frequencies 400.13 MHz for ^1^H, 100.61 MHz for ^13^C, and 79.53 MHz for ^29^Si, respectively), equipped with a 4mm dual broadband CP-MAS probe. ^13^C spectra were acquired by using the quantitative multiple CP approach described by Johnson and Schmidt-Rohr [49]. Chemical shifts were referenced externally against the carbonyl signal of glycine with δ = 176.03 ppm.

### 4.5. Scanning Electron Ricroscopy (SEM)

Electron microscopic images and EDX analyses were realized on a FEI INSPECT S50 instrument (Hillsboro, OR, USA). Samples were sputtered with gold (layer thickness of 4 nm) in a Leica Microsystems (Wetzlar, Germany) EM SCD005 sputter coater. Element mapping of silicon and sulfur was conducted with a Hitachi (Chiyoda, Tokyo, Japan) TM3000 tabletop microscope.

### 4.6. Infrared Spectroscopy (IR)

A PerkinElmer (Waltham, Massachusetts, United States) Frontier IR Single-Range spectrometer in ATR mode was used for infrared spectroscopy experiments. The spectra were measured with 4 scans per measurement between 4000 cm^−1^ and 650 cm^1^, normalized and base-line corrected before evaluation.

### 4.7. Determination of the Vinyl, Thiol and Azide Loading

The azide loading was determined by FTIR spectroscopy using a calibration curve based on a previous publication (Figure S4) [6].

The amounts of vinyl- and thiol groups on the cellulose surface were both determined based on quantitative solid-state NMR (Figures S1 and S2). This data was correlated with the IR absorbance of the Si-O band at 760 cm^−1^ in case of vinyl-modified cellulose (Figure S4) or at 804 cm^−1^ in case of thiol-modified cellulose (Figure S4) to create calibration curves for estimating the silane loading.

### 4.8. Aqueous Stability of Silanized Cellulose

The stability of the CNF-vinyl under aqueous conditions was investigated in three different buffer systems (each 50 mM), an acetate buffer at pH 4, a phosphate buffer at pH 7 and an ethanolamine buffer at pH 10. The amount of remaining silane on the cellulose surface was determined by measuring the absorbance of the Si-O band at 760 cm^−1^.

### 4.9. Gel Permeation Chromatography (GPC)

GPC measurements were carried out using the following setup: online degasser Dionex (Sunnyvale, California, United States) DG-2410; Kontron (Augsburg, Germany) 420 pump, pulse damper; auto sampler, HP 1100; column oven, Gynkotek (Munich, Germany) STH 585; MALLS detector, Wyatt (Santa Barbara, California, United States) Dawn DSP with argon ion laser (λ_0_ = 488 nm); RI detector, Showa Denko (Tokyo, Japan) Shodex RI-71. Data was evaluated with Wyatt ASTRA software. The following settings were used: flow: 1.00 mL min^−1^; columns: four Agilent Technolgies (Santa Clara, California, United States) PLgel mixed-A LS, 20 µm, 7.5 × 300 mm; injection volume: 100 µL (sample concentration: 10–20 mg mL^−1^); run time: 45 min; mobile phase: DMAc/LiCl (0.9% *w*/*v*). 

## Supplementary Materials

The following are available online, Figure S1: Quantitative solid state ^13^C-NMR spectra of mercapto samples, Figure S2: Quantitative solid state ^13^C-NMR spectra of vinyl samples Figure S3: Silanization of carboxymethylated pulp, Table S1: Silanized cellulose substrates: comparison of the IR absorbance of the significant band and silane loading of the samples, Figure S4: IR spectra of mercapto-, vinyl- and azido-functionalized samples, Figure S5: Proof of concept of availability of free thiol groups, Figure S6: IR spectra of the kinetics of the different silanes at room temperature with lyocell gel as substrate, Figure S7: Avenue to multifunctional colloids.
