# Supplementary material for: A General Aqueous Silanization Protocol to Introduce Vinyl, Mercapto or Azido Functionalities onto Cellulose Fibers and Nanocelluloses
Source: Molecules. 2018 Jun 12;23(6):1427. doi: 10.3390/molecules23061427 (PMC6100551; doi:10.3390/molecules23061427)
Supplement: Supplementary file 1 [file molecules-23-01427-s001.pdf]

Supplemental Information

A general aqueous silanization protocol to introduce vinyl, mercapto or azido functionalities onto cellulose fibers and nanocelluloses

Marco Beaumont,[a*] Markus Bacher,[a] Martina Opietnik,[b] Wolfgang Gindl-Altmutter,[c] Antje Potthast,[a] Thomas Rosenau[a,d*]

[a] University of Natural Resources and Life Sciences Vienna (BOKU), Department of Chemistry, Division of Chemistry of Renewable Resources, Konrad-Lorenz-Straße 24, A-3430 Tulln, Austria

[b] Lenzing AG, Werkstraße 2, 4860 Lenzing, Austria

[c] University of Natural Resources and Life Sciences Vienna (BOKU), Department of Materials Science and Process Engineering, Institute of Wood Technology and Renewable Materials, Konrad-Lorenz-Straße 24, A-3430 Tulln, Austria

[d]Johan Gadolin Process Chemistry Centre, Åbo Akademi University,
Porthansgatan 3, Åbo/Turku FI-20500, Finland

*Corresponding authors:

Dr. Thomas Rosenau, Tel.: +43 1 47654-77411, thomas.rosenau@boku.ac.at .
Dr. Marco Beaumont, Tel.: +43 1 47654-77436, marcobeaumont1@gmail.com .

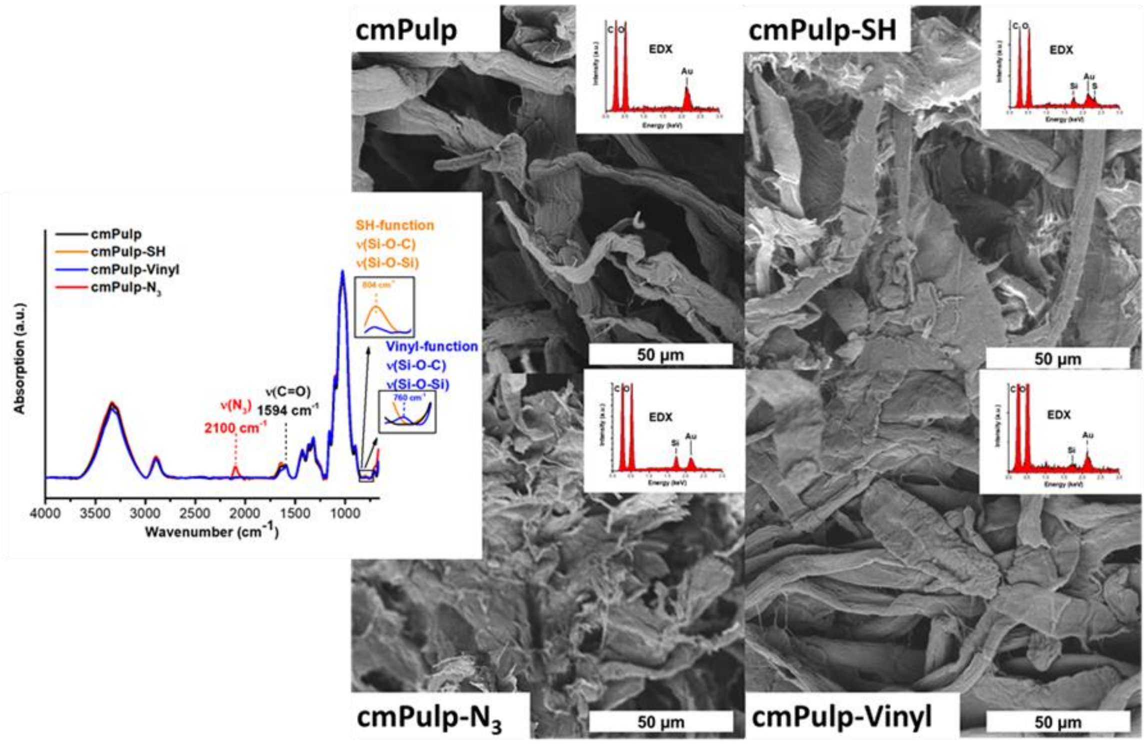

Figure S1. Silanization of carboxymethylated pulp using mercapto-, azido- and vinyl-silane as shown by IR spectroscopy, SEM-EDX and study of its morphology using SEM.

Table S1. Silanized cellulose substrates: comparison of the IR absorbance of the significant band and silane loading of the samples. Silane loading was determined from calibration curves in Figure S2. *Silane loading was not in the range of the calibration curve, *cf.* Figure S2.

	Reaction with triethoxy 3-azidopropylsilane		Reaction with triethoxy vinylsilane	
**Substrates**	*IR Absorbance (cm^{-1})*	*Silane loading (mmol/g)*	*IR Absorbance (cm^{-1})*	*Silane loading (mmol/g)*
Lyocell gel (LG)	0.26	1.21	0.09	1.18
Cellulose nanofibrils (CNF)	0.12	0.52	0.02	0.41
carboxymethylated pulp (cmPulp)	0.06	0.22	0.0003	-*

	Reaction with trimethoxy 3-mercaptopropylsilane	
**Substrates**	*IR Absorbance (cm^{-1})*	*Silane loading (mmol/g)*
Lyocell gel (LG)	0.029	0.97
Cellulose nanofibrils (CNF)	0.018	0.65
carboxymethylated pulp (cmPulp)	0.013	0.50

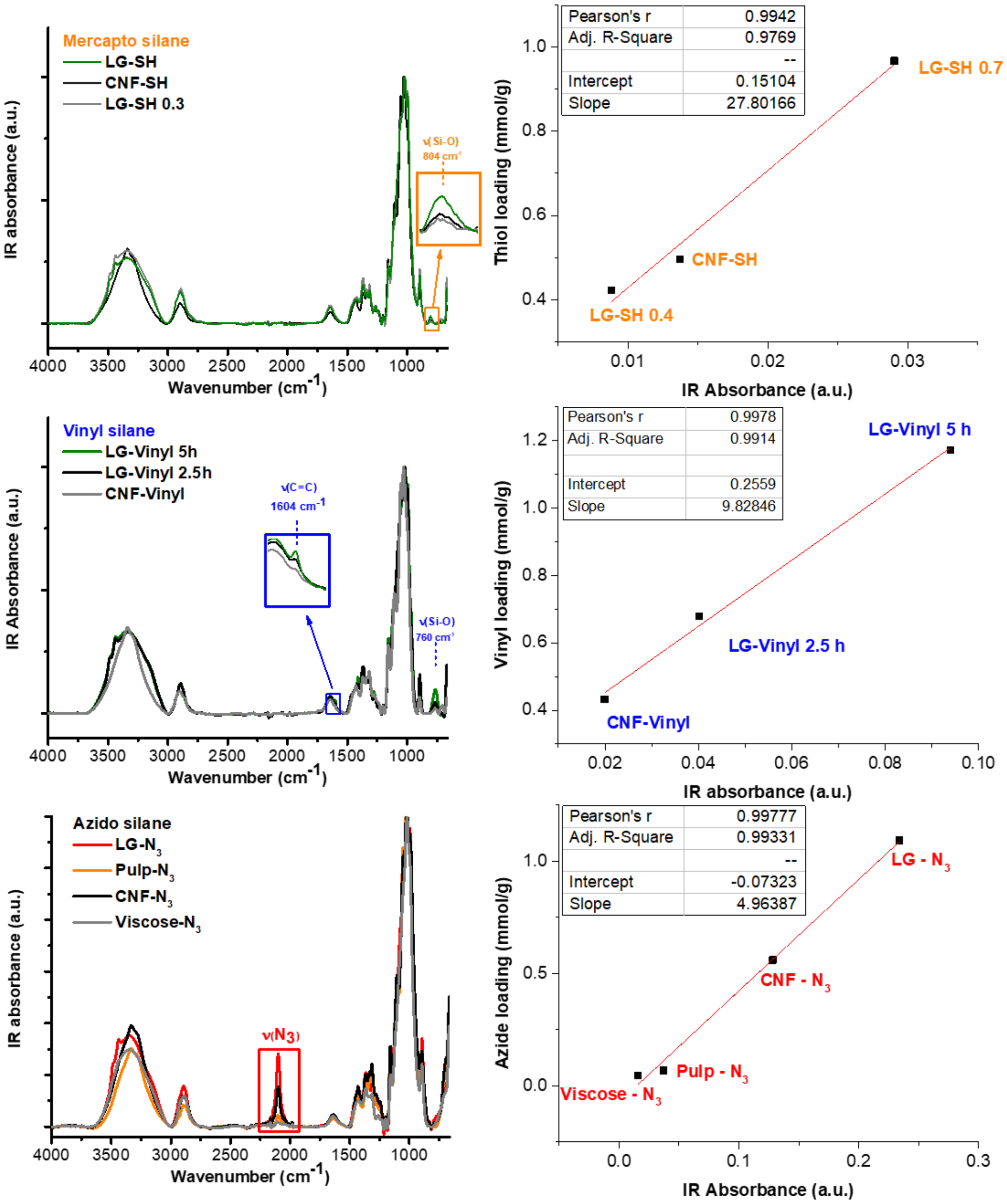

Figure S2. IR spectra of mercapto-, vinyl- and azido-functionalized samples with characteristic bands at 804 cm^{-1}, 760 cm^{-1} and 2100 cm^{-1} were used in combination with quantitative ^{13}C solid-state NMR spectra of vinyl samples from Figure S3 and mercapto samples from Figure S4 and published data of azido samples from the literature (Hettegger and Beaumont et al. 2016) to obtain calibration curves for fast and simple estimation of the silane loading using ATR-FTIR.

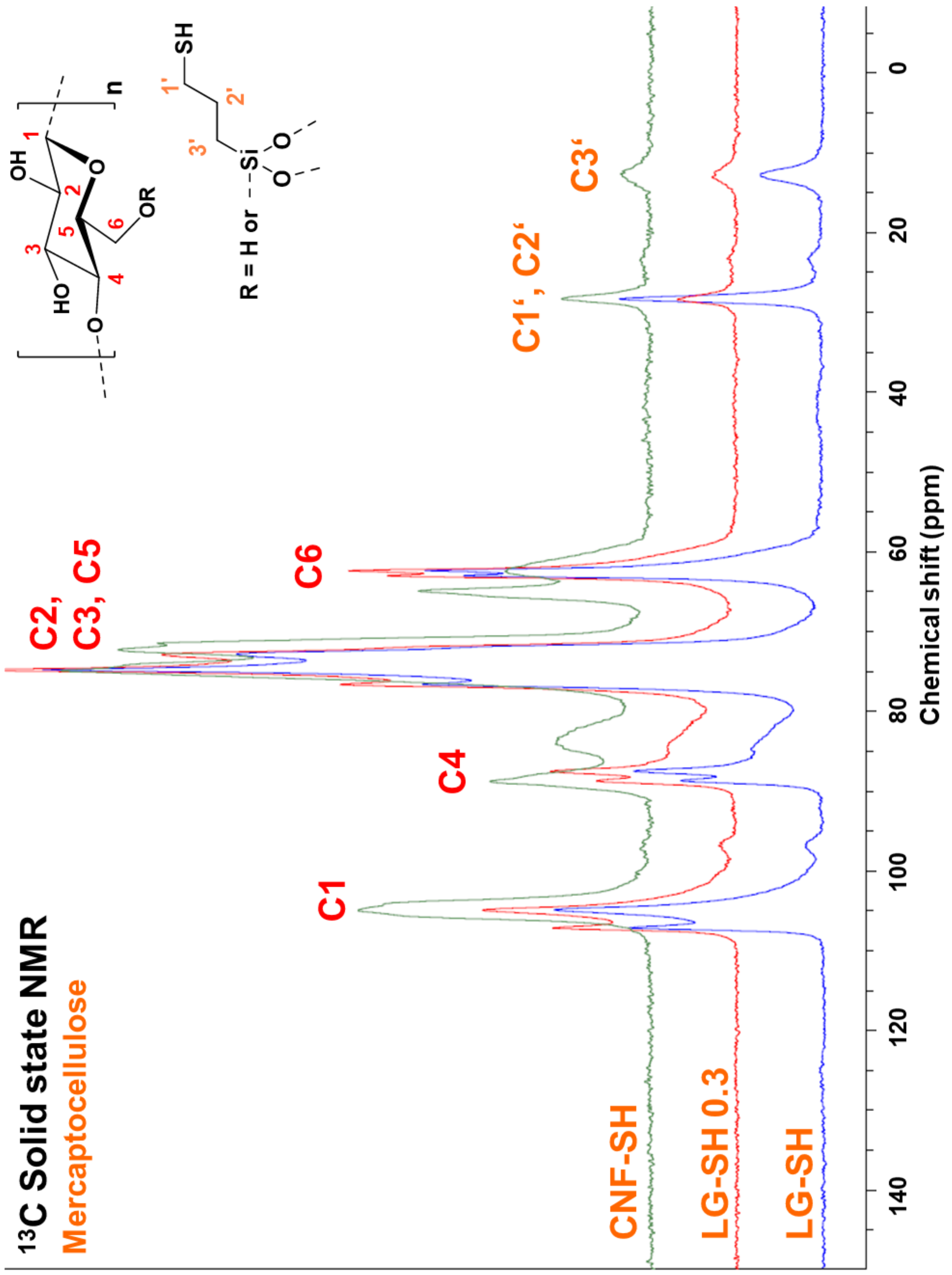

Figure S3. Quantitative solid state ^{13}C-NMR spectra of mercapto samples.

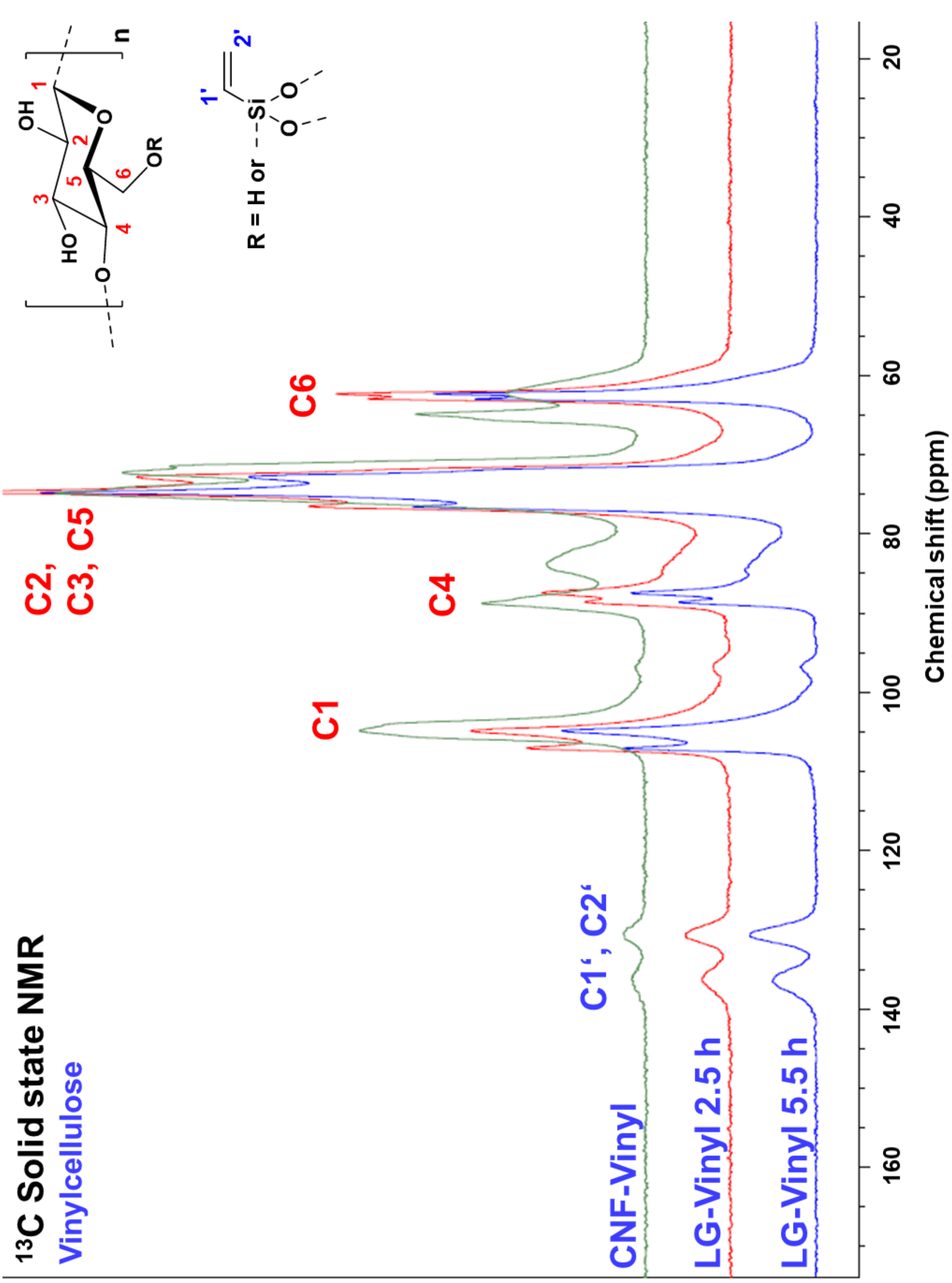

Figure S4. Quantitative solid state ^{13}C-NMR spectra of vinyl samples.

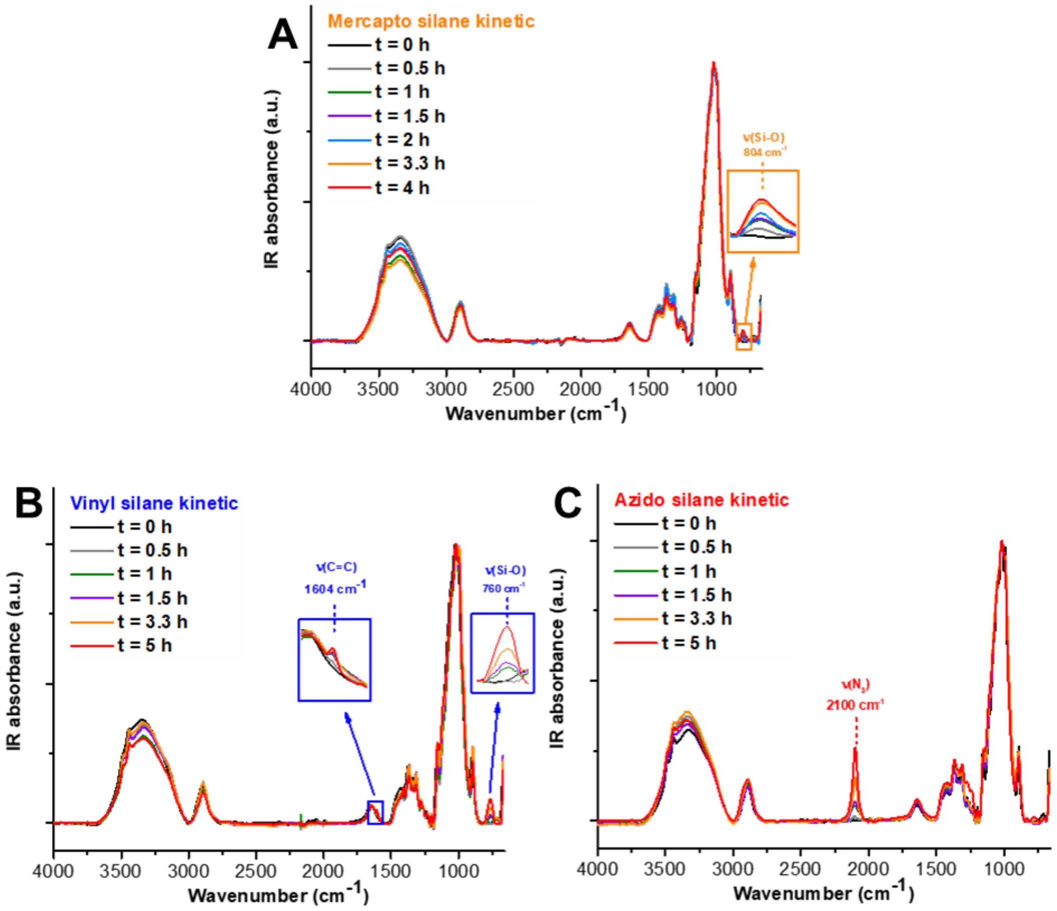

Figure S5. IR spectra of the kinetics of the different silanes at room temperature with lyocell gel as substrate.

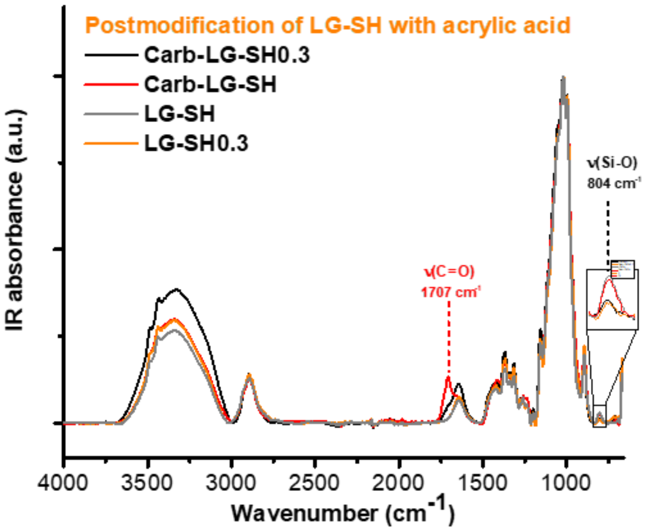

Figure S6. Proof of concept of availability of free thiol groups in case of LG-SH by radically induced thiol-ene reaction with acrylic acid. The presence of the carboxyl band at 1707 cm^{-1} proves the successful introduction of carboxyl groups.

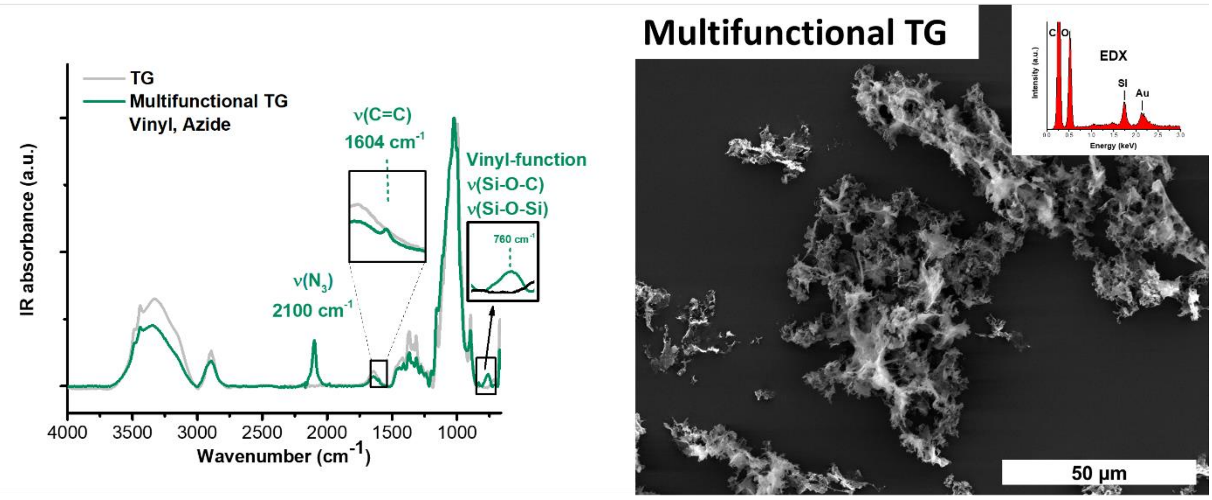

Figure S7. Avenue to multifunctional colloids by silane treatment using both, vinyl- and azido-silane.
